# The *rsmS* (*ybaM*) mutation causes bypass suppression of the RsmAB post-transcriptional virulence regulation system in enterobacterial phytopathogens

**DOI:** 10.1038/s41598-019-40970-3

**Published:** 2019-03-14

**Authors:** Rita E. Monson, Katinka Apagyi, Steven D. Bowden, Natalie Simpson, Neil R Williamson, Marion F. Cubitt, Steve Harris, Ian K. Toth, George P. C. Salmond

**Affiliations:** 1 Department of Biochemistry, Hopkins Building, Tennis Court Road, University of Cambridge, Cambridge, CB2 1QW UK; 2 Cell and Molecular Sciences, James Hutton Institute, Invergowrie, Dundee, DD2 5DA UK; 3 Present Address: Faculty of Medicine, School of Public Health, Imperial College, London, St Mary’s Campus, Norfolk Place, W2 1PG UK; 4 Present Address: Department of Food Science and Nutrition, University of Minnesota-Twin Cities, St. Paul, Minnesota USA; 5 Present Address: School of Sport, Exercise and Health Sciences, Loughborough University, Loughborough, LE11 3TU UK

## Abstract

Plant cell wall degrading enzymes (PCWDEs) are the primary virulence determinants of soft rotting bacteria such as the potato pathogen, *Pectobacterium atrosepticum*. The regulation of secondary metabolite (Rsm) system controls production of PCWDEs in response to changing nutrient conditions. This work identified a new suppressor of an *rsmB* mutation – *ECA1172* or *rsmS* (*rsmB* suppressor). Mutants defective in *rsmB* (encoding a small regulatory RNA), show reduced elaboration of the quorum sensing molecule (*N*-3-oxohexanoyl-homoserine lactone; OHHL) and PCWDEs. However, OHHL and PCWDE production were partially restored in an *rsmB, rsmS* double mutant. Single *rsmS* mutants, overproduced PCWDEs and OHHL relative to wild type *P. atrosepticum* and exhibited hypervirulence in potato. RsmS overproduction also resulted in increased PCWDEs and OHHL. Homology searches revealed *rsmS* conservation across pathogens such as *Escherichia coli (ybaM)*, *Dickeya solani, Klebsiella pneumoniae* and *Shigella flexneri*. An *rsmS* mutant of *Pectobacterium carotovorum* ATCC39048 showed bypass of *rsmB*-dependent repression of PCWDEs and OHHL production. *P. carotovorum* ATCC39048 produces the β-lactam antibiotic, 1-carbapen-2-em-3-carboxylic acid (a carbapenem). Production of the antibiotic was repressed in an *rsmB* mutant but partially restored in an *rsmB, rsmS* double mutant. This work highlights the importance of RsmS, as a conserved pleiotropic regulator of virulence and antibiotic biosynthesis.

## Introduction

Members of the *Pectobacteriaceae* are the causative agents of soft rot in potato tubers and blackleg in potato plants. These Gram-negative bacteria, such as *Pectobacterium atrosepticum* SCRI1043 (Pba) and *Pectobacterium carotovorum* (Pcc), are responsible for diminished yields due to diseased plants, seed potato infection, and soft rot decay of tubers post-harvest during storage. Both soft rot and blackleg diseases are caused by the secretion of plant cell wall degrading enzymes (PCWDEs), such as pectate lyases, polygalacturonase, cellulases and proteases^1^. Flagellum-dependent motility is also important for plant virulence^1,2^. The production and secretion of PCWDEs and other virulence determinants is tightly controlled by many interconnected regulatory networks including an acyl-homoserine lactone quorum sensing (QS) system and the regulation of secondary metabolite (Rsm) system^3–5^.

Cell density-dependent expression of PCWDEs in Pba and Pcc is regulated by the ExpI-VirR (ExpR2 in some species) QS system^3,6–8^. ExpI, an acyl-homoserine lactone synthase, produces the freely diffusible signalling molecule *N*-3-(oxohexanoyl)-homoserine lactone (OHHL) which acts as a proxy for population density, and VirR represses transcription of target genes^3^. Above a threshold concentration, OHHL interacts with VirR, relieving repression, resulting in transcription of genes encoding PCWDEs, siderophore biosynthesis, and flagellar motility^3,6,7,9^. Furthermore, in the absence of OHHL, VirR binds the promoter of *rsmA*, another important virulence regulator, activating its transcription and linking the Rsm and QS systems^9^.

The Rsm regulatory system acts post-transcriptionally to regulate translation of target mRNAs by blocking the Shine-Dalgarno site and influencing transcript stability^4,6,7^. The primary component of the system is the RNA-binding protein RsmA (CsrA in *Escherichia coli*). In *Pectobacterium* spp., RsmA interacts with transcripts for virulence determinants, thereby influencing *in planta* infection^10^. RsmA/CsrA binds to GGA motifs of specific transcripts, blocking ribosomal access, resulting in decreased translation^11,12^. RsmA-binding can be influenced by the untranslated RNA, *rsmB*^13^. When processed and folded, *rsmB* forms a multi-stem-loop structure with GGA motifs exposed at the end of each loop. Therefore, a single copy of *rsmB* can titrate many copies of RsmA simultaneously and this stoichiometry has a crucial impact on regulatory functionality^14,15^. In Pba and Pcc, an *rsmB* mutant shows constitutively active RsmA and produces almost no PCWDEs; even less than an *expI*, QS-defective, mutant^13,14,16^.

A previous study attempted to identify a mutation in *rsmA* by isolating suppressors of *rsmB*^16^. Unexpectedly, this strategy identified a mutant with a transposon insertion in *metJ* that was found to suppress the *rsmB* mutation. MetJ is a metabolic regulator known to bind DNA at *met*-boxes, often found upstream of methionine biosynthetic genes^17–20^. While there had been several studies investigating *metJ* and its role in metabolism^20–22^, Cubitt *et al*. demonstrated a link between MetJ, virulence, and the Rsm system^16^. Furthermore, a mutation in *metJ* resulted in pleiotropic transcriptional changes, suggesting that it affected multiple cellular pathways^16^. We postulated that there might be other suppressors of *rsmB*, still to be identified.

To investigate suppression of the Rsm system further, a random transposon mutagenesis of an *rsmB* mutant of Pba was conducted and insertion mutants with increased protease (caseinase) production were selected for further analysis. From this screen, transposon insertions in *priC* and *ECA1172*, a small open reading frame (ORF) of unknown function immediately downstream of *priC*, were identified. We demonstrated that suppression of *rsmB* was via mutation in *ECA1172*, a gene we named *rsmS* for *rsmB* suppressor.

## Results

### Identification of ECA1172, a new regulator of *rsmB*-dependent virulence repression

**Figure 1:**
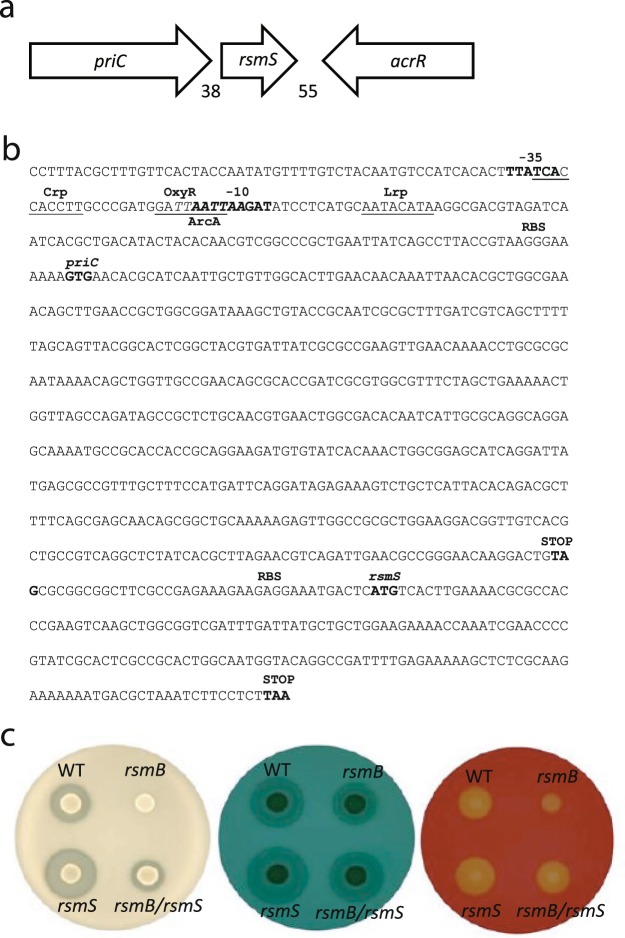
Mutation of *rsmS* affects biosynthesis of PCWDEs. (**a**) Genomic context of *rsmS* within Pba. Genes are indicated with arrows and names are written within. The numbers below indicate the number of bp between each gene. (**b**) The *priC-rsmS* promoter region. The −10 and −35 sites are indicated in bold. Putative binding sites for Crp, OxyR, and Lrp are indicated with a line under the site. A putative ArcA binding site is indicated in italics as it overlaps both the OxyR and −10 site. Putative Shine-Dalgarno sites are indicated as RBS and the translational start and stop sites are indicated in bold. (**c**) PCWDE plate assays showing production of protease (left), pectate lyase (middle) and cellulase (right) for the wild type, *rsmB* (NW155), *rsmS* (KA32) or *rsmB rsmS* (KA35) after incubation at 25 °C.

A Pba *rsmB* mutant produces no detectable protease activity^16^. To identify suppressors of this phenotype, over 20 000 random transposon insertion mutants in an *rsmB* mutant background were screened for restored protease production. A single transposon insertion 12 bp within the *priC* gene, (encoding the primosomal replication protein N), and several insertions in the ORF immediately downstream, *ECA1172* (a gene of unknown function) were identified (Fig. 1a). Each of these transposon insertions partially restored protease production (Fig. 1 and Supplemental Fig. 1). As the *ECA1172* insertion produced an *rsmB* suppressor phenotype, we named it *rsmS*. Both *priC* and *rsmS* were novel suppressors of *rsmB*-dependent repression of PCWDEs and, therefore, warranted further characterization.

### Mutation of *priC* causes polar effects on *rsmS*

Genomic analysis suggested that the *priC* and *rsmS* genes were within an operon (Fig. 1a). The two ORFs were separated by 37 bp, transcribed in the same direction, and we identified a candidate promoter upstream of *priC* but no promoter within 250 bp of the *rsmS* translational start site (Fig. 1b). The transposon used in the screen contained a transcriptional terminator^23^, thus, if *priC* and *rsmS* were in an operon, a transposon insertion within *priC*, would also affect production of the *rsmS* product. To determine if suppression of *rsmB* observed in the *priC* transposon mutant was due to a polar effect on *rsmS*, protease production was observed when a plasmid-borne copy of either *priC* or *rsmS* was expressed in a *priC* mutant. No change in production was detected when PriC was expressed from a plasmid, but protease production decreased when RsmS was expressed in a *priC* mutant background, suggesting that the mutation had been complemented (Supplemental Fig. 1). Furthermore, we had identified transposon insertions within *rsmS¸* the ORF immediately downstream, which also partially restored protease production in an *rsmB* mutant. We concluded that the two genes were transcribed as an operon and that it was the downstream effect on *rsmS* in the *priC* mutant that partially suppressed the *rsmB* mutation.

### Mutation of *rsmS* restores production of PCWDEs in an *rsmB* mutant

To exclude the possibility of a secondary mutation elsewhere in the chromosome affecting protease production, a new mutation in *rsmS* was created by allelic exchange and was transduced into an *rsmB* mutant (creating strain KA35) and into wild type Pba (creating KA32). Production of protease was still partially restored in KA35 (*rsmB, rsmS*), suggesting that it was the deletion of *rsmS* and not another mutation that was responsible for this phenotype (Fig. 1c). We continued to work with KA35 and KA32 for the remainder of this study. As we had been able to phenotypically complement a mutation in *priC* with a copy of *rsmS in trans*, we knew that this plasmid-borne copy of *rsmS* was functional. We introduced the same plasmid into KA35 (*rsmB, rsmS*) and found that protease production was no longer detectable (Supplemental Fig. 2a). We concluded that mutation of *rsmS* alone was capable of restoring protease production in an *rsmB* mutant and that expression of *rsmS in trans* complemented this phenotype.

Cellulase and pectate lyase are both important for potato infection and production of both enzymes is significantly reduced in an *rsmB* mutant^1,16^. As observed for protease production above, in an *rsmB, rsmS* double mutant, significantly more cellulase or pectate lyase activity was detected than in an *rsmB* mutant (Fig. 1c). Though enzymatic activity was not as high as in wild type Pba, an *rsmS*, *rsmB* double mutant produced more PCWDEs than a strain mutated for *rsmB* alone (Fig. 1c). At the same time, we examined strain KA32 (*rsmS*) and found that secreted enzyme levels were increased, relative to wild type (Fig. 1c). We concluded that RsmS repressed PCWDEs in Pba.

Swimming motility is an important virulence determinant in Pba^2,10,24^, thus we examined whether mutation of *rsmS* affected flagellum-dependent swimming. Using low percentage agar, we found no significant difference in swimming motility between wild type Pba, an *rsmB* mutant, an *rsmS* mutant or an *rsmB rsmS* double mutant (data not shown). This observation was consistent with other work examining *rsmB* mutant phenotypes in Pba^16^.

### An *rsmS* mutation bypasses repression of PCWDEs and OHHL in an *rsmB* mutant

**Figure 2:**
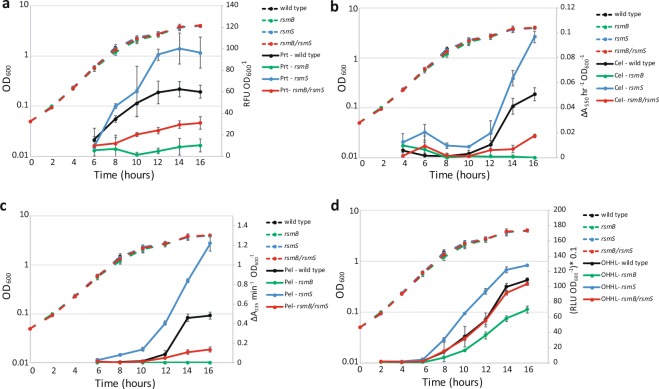
An *rsmS* mutation alters PCWDE and OHHL production. Levels of protease (**a**), cellulase (**b**), pectate lyase (**c**) and OHHL (**d**) in the culture supernatant determined at two-hour intervals throughout growth in PMB at 25 °C. Wild type (black), *rsmS* (KA32, blue), *rsmB* (NW155, green) and *rsmB rsmS* (KA35, red) cultures were assessed for growth (OD_600_ – dashed lines) and sterile supernatant samples taken every two hours. Solid lines indicate the enzyme activity or relative OHHL amounts determined from sterile supernatants. Values indicate the average +/− SD (n = 3).

To ascertain if growth rate or cell density-dependent expression of virulence factors was altered by mutation of *rsmS*, the activities of PCWDEs throughout growth in wild type, KA32 (*rsmS*), KA35 (*rsmB, rsmS*) or NW155 (*rsmB*) strains were determined. No significant differences in optical density or growth rate were observed between any of the strains, suggesting that mutation of *rsmS* caused no discernible growth defect in these media (Fig. 2). Sterile supernatant samples, taken at each time point, were analysed for cellulase, pectate lyase or protease activity. Mutants defective in *rsmB* showed reduced levels of all enzymes compared to those in wild type Pba. Supernatants taken from strain KA35 (*rsmB, rsmS*) showed significantly greater enzyme activity than those observed in an *rsmB* mutant (Fig. 2). These data were consistent with observations on plates and in the original screen: a mutation in *rsmS* phenotypically suppressed the effects of an *rsmB* mutation.

We found that mutation of *rsmS* in an otherwise wild type background resulted in significantly increased secreted enzymes but no growth defect, reinforcing the findings of earlier plate-based assays (Figs 1 and 2). For example, after 16 hours of growth, secreted pectate lyase activity of an *rsmS* mutant was 2.5-fold higher than that of wild type Pba (Fig. 2c). Similar patterns were observed for cellulase and protease production (Fig. 2a,b). These data, together with plate-based assays, suggested that, although *rsmS* was originally identified as a suppressor of *rsmB* in double mutants, an *rsmS* mutation may be sufficient to cause hypervirulence (Fig. 2).

### Mutation of *rsmS* alters levels of the QS signalling molecule OHHL

An *rsmB* mutant of Pba produces reduced amounts of OHHL^16^. We wanted to determine whether mutation of *rsmS*, in an *rsmB* mutant background, perturbed OHHL production, as this would influence production of PCWDEs. The same sterile supernatant samples (see previous section) were assessed for the presence of OHHL using a bioluminescent reporter assay in *Escherichia coli*^25^. In an *rsmB, rsmS* double mutant, production of OHHL was indistinguishable from that of wild type (Fig. 2d). Therefore, in addition to suppressing the effects of *rsmB* on production of virulence determinants, an *rsmS* mutation completely relieved *rsmB*-dependent repression of OHHL production. In the *rsmS* mutant, OHHL biosynthesis was significantly greater than that observed in wild type cultures (Fig. 2d), suggesting that RsmS was also a repressor of OHHL production (and thus QS).

### Overexpression of *rsmS* resulted in increased enzyme secretion

The plasmid-based expression system (pBAD30) used for complementing protease production in an *rsmS* mutant, provided *rsmS* on a plasmid under the control of the P_ara_ promoter (Supplemental Fig. 2). Expression from this promoter is induced by increasing arabinose concentrations^26^. Using pBAD30-*rsmS*, we assessed protease production when *rsmS* was overexpressed in wild type Pba, an *rsmB* mutant, an *rsmB, rsmS* double mutant or an *rsmS* mutant. In all strains, overexpression of *rsmS* led to an increase in secreted protease activity, though again this was difficult to quantify on a plate (Supplemental Fig. 2). Surprisingly, even overexpression of *rsmS* in an *rsmB* mutant resulted in increased protease production (Supplemental Fig. 2). Therefore, these results showed that both loss of RsmS or an excess of RsmS resulted in increased secreted protease.

**Figure 3:**
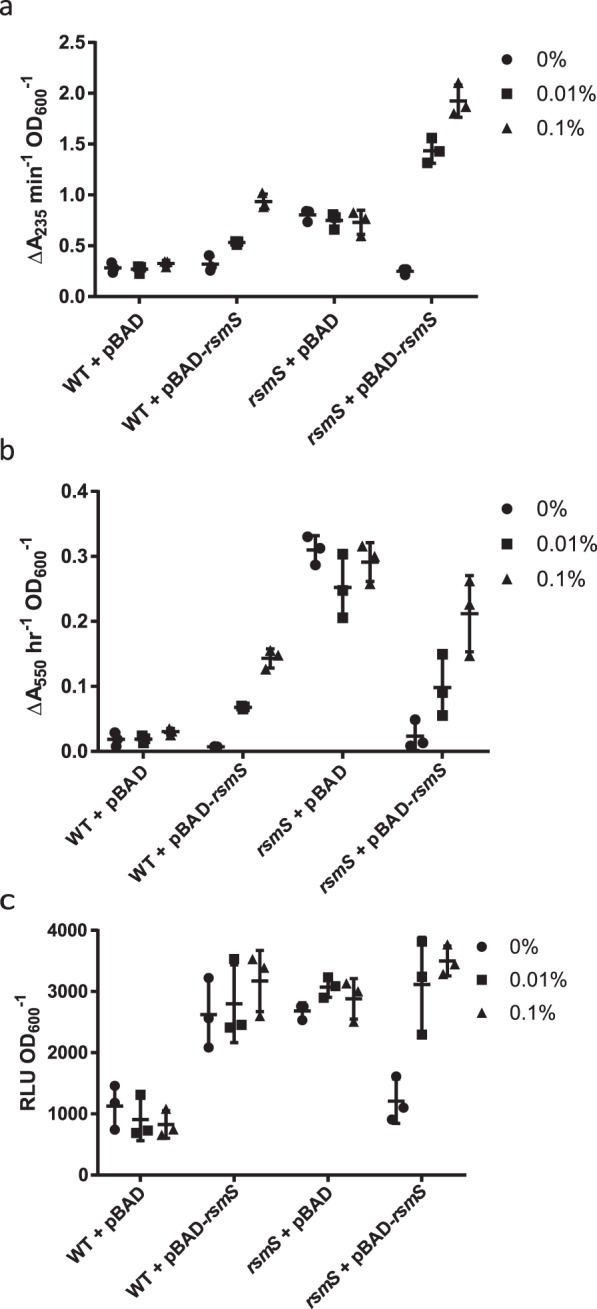
Perturbation of RsmS alters production of PCWDEs and OHHL. Pectate lyase (**a**), cellulase (**b**) or OHHL (**c**) production in wild type Pba (WT) or an *rsmS* mutant (KA32) carrying either pBAD30 or pBAD-*rsmS* grown in PMB with 0% arabinose (circle), 0.01% arabinose (square) or 0.1% arabinose (triangle) for 16 hours at 25 °C. Plots all show individual data points, the mean and error bars indicate +/− SD (n = 3).

To investigate whether increased RsmS altered other virulence-associated phenotypes, we examined pectate lyase, cellulase and OHHL production in sterile culture supernatants from wild type Pba or an *rsmS* mutant carrying either pBAD30 (empty vector) or pBAD30-*rsmS* under increasing levels of induction. In wild type cultures, even in uninduced conditions, all three phenotypes were elevated when RsmS was expressed from a plasmid, compared to the empty vector control (Fig. 3a–c). As production of RsmS was induced, a progressive increase in cellulase and pectate lyase activity was observed (Fig. 3a,b). In contrast, OHHL levels increased when an extra copy of *rsmS* was present on a plasmid but did not change upon RsmS induction. These data suggested that, in wild type Pba, even a low level of *rsmS* transcription, through leaky expression and without induction, resulted in maximal OHHL production (Fig. 3c). In an *rsmS* mutant containing pBAD30-*rsmS* under uninduced conditions, reduced levels of OHHL, pectate lyase and cellulase were observed. These phenotypes were indistinguishable from those of wild type Pba, indicating that the mutation had been complemented. However, when RsmS was induced, levels of cellulase, pectate lyase and OHHL increased back to those observed in an *rsmS* mutant without induction (Fig. 3). Together these results showed that the relative abundance of RsmS in the cell, and not just its absence, impacted the production of PCWDEs and OHHL.

### Mutation of *rsmS* results in increased potato rot

**Figure 4:**
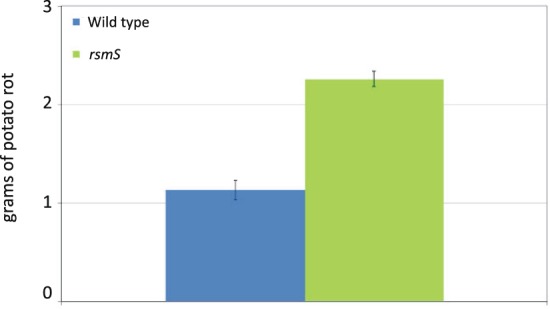
Mutation of *rsmS* alters *in planta* virulence. Values plotted represent the average amounts of potato rot (+/− SD) harvested from wild type (blue) and *rsmS* (KA32, green) inoculations after four days of incubation at 25 °C from four potato tubers.

Pba causes tuber rot^1^. Our earlier results demonstrated that mutation of *rsmS* resulted in increased production of PCWDEs (Fig. 2). Consequently, we assessed *in planta* virulence of this strain. Surface sterilized potatoes were inoculated with wild type Pba and an *rsmS* mutant at two inoculation sites on opposite sides of the potato. After four days of growth, the level of rot was measured at the inoculation sites. The weight of rot at the *rsmS* inoculation site was significantly larger than at the wild type site (Fig. 4), confirming our earlier results that the *rsmS* mutant was hypervirulent.

### RsmS is conserved across many model organisms, though its genomic context can differ

**Figure 5:**
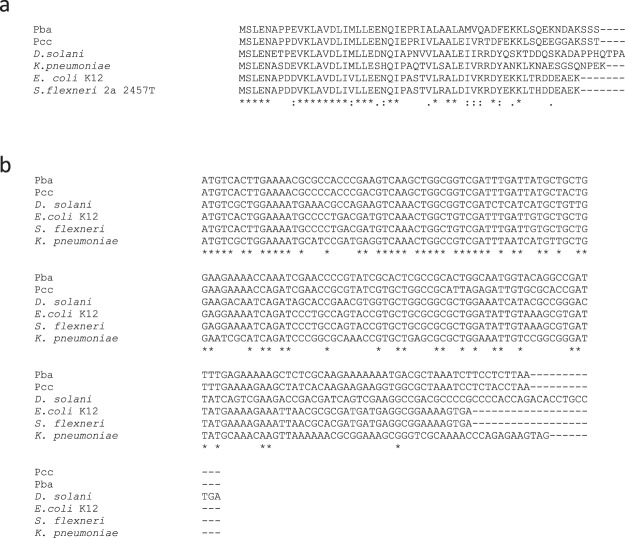
RsmS is conserved in different bacteria. Amino acid (**a**) and nucleotide (**b**) alignments of RsmS or *rsmS* from Pba, Pcc39048, *D. sol*an*i* MK10, *K. pneumoniae*, *E. coli* K 12 and *S. flexneri* 2a 2457 T. In (**a**) positions conserved across all sequences are indicated with an * below, amino acids that share strongly similar properties across all sequences are indicated with a :, and a . indicates amino acids that share weakly similar properties across the sequences. (**b**) Nucleotides that are conserved across all sequences are indicated by * and nucleotides that did not align and represent a gap are indicated with a -.

**Figure 6:**
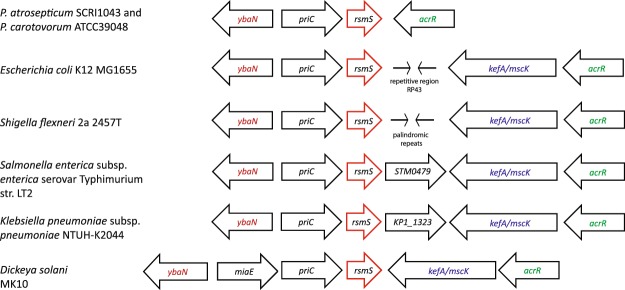
Genomic contexts of *rsmS* in different bacterial species. Arrows are used to indicate each ORF in the surrounding area of *rsmS* with the ORF name inside. Red arrows indicate *rsmS* or its homologue in each organism. ORFs that are similar are indicated in the same colour of text. Repeat regions are indicated with stick arrows.

Given the importance of RsmS in regulating plant virulence in Pba and the widespread presence of the Rsm/Csr regulation system across different bacterial species^4,27,28^, we determined whether *rsmS* was also widely distributed. We identified *rsmS* homologues (or *ybaM* as it is designated in *E. coli*) across many different bacteria and found that the gene was always immediately downstream of *priC* (Figs 5 and 6). The predicted size of RsmS_Pba_ was 6.1 kDa and, using Pfam^29^, we were able to identify a single conserved domain pfam10689 spanning amino acids 2–44. However, this domain has no known function. We aligned the RsmS amino acid sequences from many different bacteria and found that, at the amino acid level, the sequence had been conserved, especially in the region covered by the pfam10689 domain (Fig. 6). Additionally, a Shine-Dalgarno sequence was identified immediately upstream of *rsmS*, suggesting that the ORF can be translated (Fig. 1). We examined the promoter region of *priC* in Pba to determine whether there were any known regulator binding sites in this area. We identified putative −10 and −35 sites and potential binding sites for the regulators Crp, OxyR, ArcA and Lrp, suggesting that some, or all, of these regulators may influence transcription of *priC*, and thus, *rsmS* (Fig. 1b).

### Mutation of *rsmS* is also capable of suppressing an *rsmB* mutant in Pcc39048

**Figure 7:**
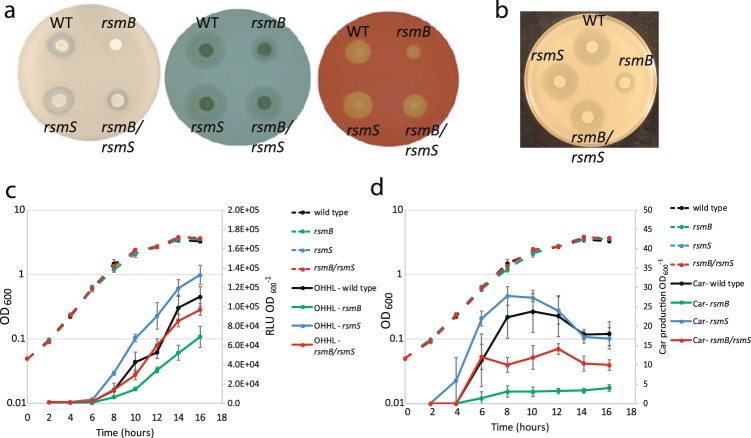
Mutation of *rsmS* affects virulence in Pcc39048. (**a**) Protease (left), pectate lyase (middle) and cellulase (right) of wild type, *rsmB* (SBEB), *rsmS* (SBES) and *rsmB rsmS* (SBEBS) mutants. Production of each enzyme is indicated by a halo surrounding the strain spot. (**b**) Carbapenem production of wild type, *rsmB*, *rsmS* and *rsmB, rsmS* double mutants. OHHL (**c**) and Carbapenem production (**d**) in Pcc39048 of wild type (black), *rsmB* (green), *rsmS* (blue) and *rsmB, rsmS* (red) throughout growth in PMB at 30 °C. Strains were monitored for optical density (Dashed line, OD_600_) and supernatant samples were examined for OHHL and carbapenem activity (solid lines). Plotted points indicate the average value of each data point +/− SD (n = 3).

Our initial focus had been the Pba Rsm system, however, in the related phytopathogen Pcc, extensive research has been undertaken to understand the Rsm system^4,13,14,30^. We were particularly interested in the strain Pcc39048, as it produces a simple carbapenem antibiotic^31^. Pcc39048 contained homologues of *rsmS* (Fig. 5) and *rsmB*, though neither had been investigated. To isolate a mutant of *rsmB*, random transposon mutagenesis of Pcc39048 was first undertaken and colonies were screened for decreased protease (caseinase) production. A transconjugant containing a transposon insertion within *rsmB* was identified using this method. The transposon insertion was transduced back into wild type Pcc39048, generating strain SBEB. The resulting transductants exhibited no protease production and reduced cellulase and pectate lyase production (Fig. 7a).

A second transposon mutagenesis was performed on the *rsmB* mutant (SBEB) in Pcc39048, to identify random transposon insertions capable of restoring caseinase production. Over 14 000 colonies were screened and two showing increased protease activity relative to the progenitor strain (SBEB) were chosen for further analysis. One of these transposon insertions was within *hexY (rsmC)*. Mutations in *hexY* have been identified previously, and are known to bypass the Rsm system^16,32,33^. The second transposon insertion was within a small ORF encoding a protein showing 82% amino acid identity to RsmS from Pba (Fig. 6a). The surrounding genomic location was the same in Pba and Pcc39048 (Fig. 5). The transposon in *rsmS* was transduced back into the *rsmB* mutant and into the wild type background. We examined these strains for pectate lyase, cellulase and protease activity and found that mutation of *rsmS* phenotypically suppressed the effects of the *rsmB* mutation (Fig. 7a). These data confirmed that RsmS was conserved between Pcc39048 and Pba and had the same function in both bacterial species.

PCWDE expression in Pcc39048 is also under QS control^3,34^ and our experiments in Pba suggested that mutation of *rsmS* affected OHHL levels. In Pcc39048, we found that OHHL production was reduced in an *rsmB* mutant but partially restored in an *rsmB, rsmS* double mutant (Fig. 7c). In an *rsmS* mutant, OHHL levels were slightly higher than in wild type, though not significantly different.

### Carbapenem antibiotic production is affected by both *rsmB* and *rsmS*

Carbapenem production is under QS control in Pcc39048^35^. As the Rsm system affected production of OHHL, it was expected that *rsmS* and *rsmB* might also affect carbapenem production. Carbapenem activity was assessed as growth inhibition surrounding a test culture on an *E. coli* super sensitive (ESS) top lawn. Mutants of *rsmB* showed reduced haloes of inhibition and an *rsmB, rsmS* double mutant showed wild type carbapenem biosynthesis. No significant difference in antibiotic production was observed between the *rsmS* mutant and wild type (Fig. 7b). To gain information on carbapenem production throughout growth, sterile supernatant samples taken at regular time intervals were assayed for antibiotic activity and compared to those of wild type. In an *rsmB* mutant, carbapenem production was less than that of wild type throughout growth. This was partially restored in an *rsmB, rsmS* double mutant. Carbapenem production was slightly increased and activity was observed earlier in the growth curve in an *rsmS* mutant, following a similar pattern to that observed for PCWDEs in Pba (Fig. 7d).

## Discussion

This work describes the identification and characterization of RsmS, a novel input to the Rsm system in both Pba and Pcc39048. Using a previously successful *rsmB* suppression screen^16^, several transposon insertions within the *priC-rsmS* operon were identified. The transposon used contained a transcriptional terminator, therefore insertions within *priC* also knocked out *rsmS* by polarity. Furthermore, we were only able to complement a *priC* insertion with *rsmS*, consistent with the operonic organization (Supplemental Fig. 1). This genetic organization is conserved across many different bacterial species, including *E. coli*. The one exception is another potato pathogen, *D. solani*, where an additional ORF, *miaE*, is found upstream of *priC*, perhaps forming a single *miaE-priC-rsmS* operon (Fig. 6). Furthermore, a study in *E. coli* examining knockouts of small proteins, found that mutation of *ybaM* (a homologue of *rsmS*) required the promoter upstream of *priC* for complementation^36^.

Given our identification of an insertion mutation in *priC*, it appears that it is not essential in Pba. More importantly, as *priC* and *rsmS* appear to be operonic, they are likely coregulated in many different bacterial species. As our bioinformatic interrogation identified, several conserved candidate regulator-binding sites were identified in the *priC* promoter region, including for Crp, OxyR and ArcA (Fig. 1). The impact of each of these regulators remains to be investigated, but the presence of putative binding sites suggests that this *priC-rsmS* operon is highly regulated in response to different environmental and nutritional inputs.

Small proteins such as RsmS/YbaM have been understudied^37,38^. However, proteomic techniques such as ribosome profiling have facilitated the identification of small proteins in a systematic way and research has begun to examine their roles in microbial physiology^39–41^. Some of the most well studied small proteins regulated sporulation in *Bacillus subtilis*, such as Sda, SpoVM and CmpA. Sda inhibits the kinase activity of KinA, part of the phosphorelay system that regulates sporulation, in response to DNA damage^42,43^. Another well studied small protein is AcrZ, a small *E. coli* protein associated with the AcrAB-TolC efflux pump^43^. Mutants defective in *acrZ* are more sensitive to tetracycline, puromycin and chloramphenicol but not all antibiotics transported by the AcrAB-TolC efflux pump, suggesting that this small protein alters the specificity of the pump^44^. Here we identified a new small protein RsmS: an important regulator of virulence and antibiotic production in *Pectobacterium*. With their varied roles in the cell and importance in drug resistance, virulence and other physiological impacts, the biology of small proteins clearly deserves further study and interrogation.

In Pba and Pcc, the QS system is also an important regulator of virulence factor production. Previous work demonstrated that OHHL production was significantly reduced in an *rsmB* mutant^13,16^. Addition of exogenous OHHL does not restore PCWDE production in an *rsmB* mutant, suggesting that the mutation does not act solely through the QS system^16^. During this work, we found that OHHL production was partially restored in an *rsmB, rsmS* mutant and that precocious OHHL production was observed in an *rsmS* mutant in both Pba and Pcc39048. Other studies have observed that addition of excess OHHL alone does not generally induce PCWDEs precociously^3,45,46^. Thus, although mutation of *rsmS* restored OHHL production in an *rsmB* mutant background, this is unlikely to be responsible for all the phenotypic changes observed. It is interesting in this context that a study investigating ppGpp-dependent regulation of PCWDEs in Pba also identified a mutation in *rsmS* (then referred to as *ECA1172*) as one that was capable of bypassing virulence suppression in a *relA/spoT* mutant background^46^.

While *rsmS*, or its homologues in other organisms, has not been examined extensively, altered *ybaM (rsmS)* transcription has been observed as part of transcriptomic studies investigating unrelated phenotypes. For example, under simulated microgravity in *E. coli, ybaM* transcript abundance was significantly increased^47^. Work examining changes in the *E. coli* commensal transcriptome during intestinal inflammation or colitis in mice found that *ybaM* mRNAs decreased 2.2-fold in IL10(−/−) mice, those suffering from colonic inflammation, compared to wild type mice^48^. Another study (mentioned above) created a *ybaM* mutant as part of an effort to systematically examine *E. coli* small proteins of unknown function. That work reported that a mutation of *ybaM* resulted in a competitive disadvantage compared to wild type *E. coli* when exposed to cell envelope stress, though this was not investigated further^36^. Notably, a *csrA* mutant is also more susceptible to cell envelope stress, though the *priC*-*rsmS* locus has not been identified as a binding target of CsrA^12^. In the present work, widespread changes in virulence phenotypes upon *rsmS* deletion or overexpression were observed. Taken together with the studies from *E. coli*, it is likely that *rsmS* is an important part of enterobacterial/pectobacterial stress response regulons and worthy of further interrogation in different model organisms.

Several studies have identified, indirectly, links between YbaM and the *E. coli* Csr regulatory system. For example, the half-life of *ybaM* is significantly reduced in a *csrA51* mutant and the abundance of *priC* and *ybaM* mRNAs are halved in a *csrD* mutant - another component of the Rsm/Csr regulatory system^27^. Perhaps most interesting, given our work in Pba, is the experimental evidence suggesting YbaM and CsrA interact. This was found as part of a report establishing the complete protein-protein interaction network in *E. coli* using yeast two-hybrid system analysis^49^. Though the finding has not been validated using complementary techniques, it suggests a connection exists between YbaM/RsmS and the Csr/Rsm regulatory systems.

**Figure 8:**
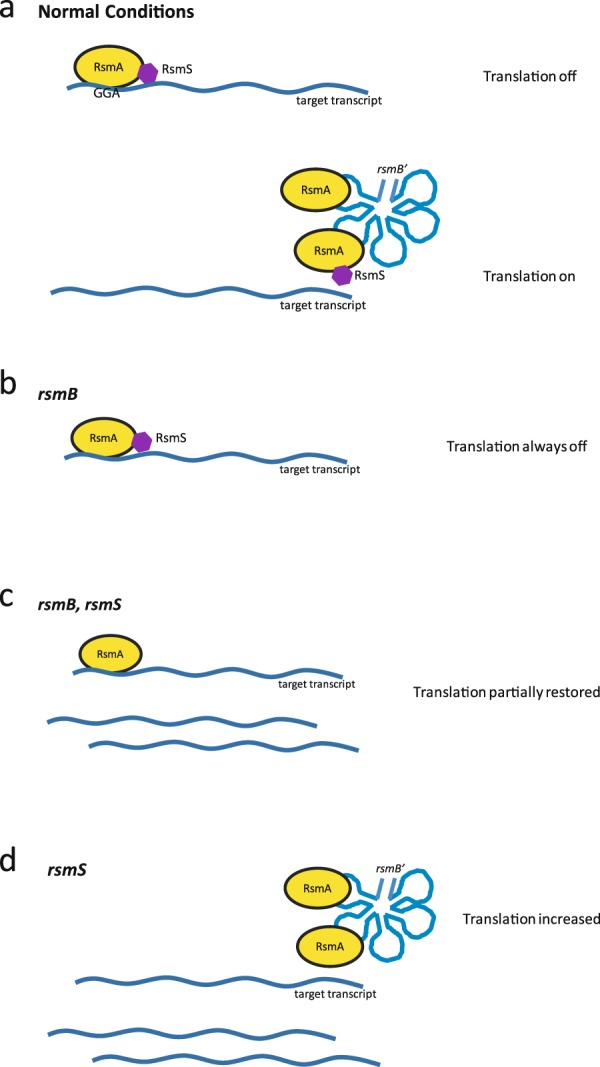
Speculative model for the function of RsmS in the Rsm regulatory system. (**a**) Under normal conditions, when *rsmB* is not present, RsmA, facilitated by RsmS, binds target transcripts, halting or slowing translation. When the untranslated regulatory RNA *rsmB* is present, it titrates RsmA away, allowing translation and production of virulence factors. (**b**) In an *rsmB* mutant, RsmA, facilitated by RsmS, is efficiently bound to any target transcripts, shutting down translation. **(c)** In an *rsmB, rsmS* double mutant, RsmA is still able to bind target transcripts, though not as efficiently as previously, leading to a partially restored translation of virulence factors. (**d**) In an *rsmS* mutant, RsmA preferentially binds rsmB over target transcripts, increasing translation of target transcripts.

Taking our experimental results together with published literature, we propose the following tentative model in Pba (Fig. 8). Under normal conditions, in the absence of *rsmB*, RsmA binds to target transcripts blocking or activating their translation. When *rsmB* is transcribed, it can titrate many copies of RsmA, ultimately leading to expression of PCWDEs. In the absence of *rsmB*, no competition exists for RsmA and it can continually repress expression of PCWDEs. However, when a mutation in *rsmB* is combined with one in *rsmS*, partial restoration of PCWDEs is observed, suggesting that RsmA binding in this strain background may be less efficient. Finally, in a strain mutated in *rsmS* alone, precocious induction of OHHL and increased PCWDEs are observed. One possibility is that RsmS is required for efficient RsmA binding to target transcripts but not essential for binding of *rsmB*. Thus, if RsmS is absent or is in excess, RsmA is not able to efficiently access its binding site on a particular transcript, favouring binding to *rsmB*. Another possible explanation is that RsmS affects the stability of RsmA, targeting it for degradation. Alternatively, RsmS may act via an undiscovered pathway, and this remains to be investigated further. For example, comparing the stability of the *rsmA* transcript in wild type versus an *rsmS* mutant might determine whether the *rsmA* mRNA is targeted for degradation. Conversely, examining RsmA protein levels in an *rsmS* mutant would determine whether this mutation perturbs the protein’s stability. Finally, as dose-dependency of RsmS is important, it would also be interesting to examine any interaction between RsmS and RsmA *in vitro*. In summary, this study has conclusively demonstrated a link between RsmS, virulence, and antibiotic production in both Pba and Pcc39048. Given the evidence from other work in *E. coli*, we think it likely that RsmS/YbaM may be involved in the stress response in many different bacterial species, reflecting a widespread physiological importance.

## Methods

### Bacterial strains and culture conditions

All bacterial strains, plasmids and bacteriophages used in this study are listed in Table 1. Unless otherwise indicated, bacterial strains were grown in Lysogeny broth-Lennox (LB, 10 g l^−1^ tryptone, 5 g l^−1^ NaCl, 5 g l^−1^ yeast extract) or Pel Minimal Broth (PMB, 0.1% (w/v) yeast extract, 0.1% (w/v) (NH_4_)_2_SO_4_, 1 mmol l^−1^ MgSO_4_, 0.5% (v/v) glycerol, 0.5% polygalacturonic acid, 40 mmol l^−1^ K_2_HPO_4_, 15 mmol l^−1^ KH_2_PO_4_, pH 7), supplemented with 1.5% agar (LBA) when in plates. *E. coli* strains were grown at 37 °C, Pcc39048 at 30 °C and Pba at 25 °C. Overnight cultures were grown in 30 ml sealed plastic universals on a roller wheel and aerated growth cultures contained 25 ml of culture in a 250 ml flask, shaken at 215 revolutions per minute (rpm). Optical density (OD_600_) was monitored using a Unicam Helios spectrophotometer. Where appropriate, antibiotics and supplements were added to media at the following concentrations: ampicillin (Ap), 50 µg ml^−1^; streptomycin (St), 50 µg ml^−1^; chloramphenicol (Cm), 50 µg ml^−1^; kanamycin (Kn), 15 µg ml^−1^ (*E. coli* strain β2163) or 50 µg ml^−1^ (otherwise); and diaminopimelic acid (DAPA), 0.3 mM.

**Table 1:** Strains, plasmids and bacteriophages used in this study.

Strain	Genotype	Reference
***Pectobacterium atrosepticum***		
SCRI 1043	Wild type	^64^
NW155	*rsmB*::*aph* Kn^R^	^16^
MC3	*rsmB::aph priC*::TnDS1028 Kn^R^ Cm^R^	This study
KA32	*rsmS*::*cat* Cm^R^	This study
KA35	*rsmB*::*aph* *rsmS*::*cat* Kn^R ^Cm^R^	This study
***Pectobacterium carotovorum***		
ATCC39048	Wild type	American Type Culture Collection
SBEB	*rsmB*::Tn-*cat* Cm^R^	This work
SBES	*rsmS*::Tn5 Kn^R^	This work
SBEBY	*hexY*::Tn5, *rsmB*::TnDS1028 Cm^R^ Kn^R^	This work
SBEBS	*rsmB*::Tn-*cat*, *rsmS*::Tn5 Cm^R^ Kn^R^	This work
***Escherichia coli***		
ESS	β-lactam antibiotic sensor strain	^57^
β2163	F- RP4-2-Tc::Mu, *dapA*::(*erm-pir*)	^65^
JM109 + pSM401	OHHL sensor strain	^25^
***Chromobacterium violaceum***		
CV026	*cviI*::Tn5; Acyl homoserine lactone indicator strain	^66^
**Bacteriophages**		
φM1	Pba generalized transducing phage	^52^
φKP	Pcc39048 generalized transducing phage	^53^
**Plasmids**		
pBAD30	Vector containing the *araBAD* promoter Ap^R^	^26^
pBAD30-*priC*	*priC* coding sequence from SCRI 1043 in pBAD30 Ap^R^	This study
pBAD30-*rsmS*	*rsmS* coding sequence from SCRI 1043 in pBAD30 Ap^R^	This study
pBluescript II SK+	*blaM*, *lacZ* Ap^R^	Stratagene
pBluescript-Δ*rsmS*	Regions surrounding *rsmS* from SCRI 1043 in pBluescript	This study
pBluescript-Δ*rsmS*-Cm	*cat* gene in *Bgl*II site of pBluescript-Δ*rsmS*	This study
pACYC184	Vector containing *cat* gene, Cm^R^	NEB
pKNG101	Allelic exchange vector St^R^	^50^
pKNG101-Δ*rsmS*-Cm	*rsmS*::Cm^R^ knockout cassette in pKNG101 St^R^	This study
pDS1028	Transposon containing plasmid used for random mutagenesis	^23^

^R^Indicates resistance.

### Bacterial strain and plasmid construction

A mutation of *rsmS* was constructed by allelic exchange^50^. The upstream and downstream regions of *rsmS* were amplified by PCR using oligonucleotide pairs oKA33/oKA34 and oKA35/oKA36 respectively (oligonucleotides used in this study are listed in Table 2). A *Bgl*II site was artificially introduced within oKA34 and oKA35. The two PCR products were then used to perform overlap PCR generating a construct containing the *rsmS* flanking regions with the introduced *Bgl*II site between them. This construct was digested with *Bam*HI/*Xho*I and ligated with compatibly digested pBluescript to create pBluescript-*ΔrsmS*. Oligonucleotides oKA42/oKA43 were then used to amplify the *cat* gene from pACYC184. This fragment and pBluescript-Δ*rsmS* were digested with *Bgl*II and ligated to create pBluescript-Δ*rsmS*-Cm. This plasmid was sequenced, digested with *Bam*HI/*Xho*I, and ligated with compatibly digested pKNG101 to create pKNG101-*ΔrsmS*-Cm. This construct was introduced into *E. coli* strain β2163 by transformation and a biparental mating set up with Pba and the *E. coli* donor cells on LBA + DAPA. The mating patches were incubated at 25 °C overnight and resuspended in one ml of LB. Serial dilutions of the mating patch were plated onto LBA + Cm + St. Double crossover mutants were identified by selection on LBA + Cm containing sucrose^50^. Colonies were subsequently screened by PCR and sequenced to confirm that the *rsmS* gene has been removed and replaced with the chloramphenicol acetyltransferase gene.

**Table 2:** Oligonucleotides used in this study.

Name	Sequence (5′-3′)	Details
oKA17	GGGGAATTCCGAGAAAGAAGAGGAAATG	Upstream oligo pBAD30-*rsmS*
oKA28	GGGAAGCTTTTAAGAGGAAGATTTAGCGTC	Downstream oligo pBAD30-*rsmS*
oKA33	GGGGATCCTGGCACTTGAACAACAAATTAACACGC	Creation of *rsmS*::Cm^R^
oKA34	CCATTGCCAGTGCGGCGAGTGAGATCTCGATACGGGGTTCGA TTTGG	Creation of *rsmS*::Cm^R^
oKA35	TCGAGATCTCACTCGCCGCACTGGCAATGG	Creation of *rsmS*::Cm^R^
oKA36	GGGGGGCTCGAGACAGATACTGGATACTGCGTT	Creation of *rsmS*::Cm^R^
oKA42	ACAGATCTGGCTATTTAACGACCC	Creation of *rsmS*::Cm^R^
oKA43	TAAGATCTCGGAAGATCACTTCGCAG	Creation of *rsmS*::Cm^R^
oKA89	GGGGAATTCCTACACAACGTCGGCCCGCTG	Upstream oligo pBAD30-*priC*
oKA91	GGAAGCTTCTACAGTCCTTGTTCCCGGCGTTCAATC	Downstream oligo pBAD30-*priC*
oMAMV1	GGAATTGATCCGGTGGATG	Sequencing oligo TnKRCPN1
oMAMV2	GCATAAAGCTTGCTCAATCAATCAC	Sequencing oligo TnKRCPN1
oPF106	GACCACACGTCGACTAGTGCNNNNNNNNNNAGAG	Random primed PCR oligo 1
oPF107	GACCACACGTCGACTAGTGCNNNNNNNNNNACGCC	Random primed PCR oligo 2
oPF108	GACCACACGTCGACTAGTGCNNNNNNNNNNGATAC	Random primed PCR oligo 3
oPF109	GACCACACGTCGACTAGTGC	Random primed PCR adaptor oligo
oREM7	CTAGAGTCGACCTGCAGGC	Sequencing oligo TnDS1028
oREM8	CACAGGAACACTTAACGGC	Sequencing oligo TnDS1028

Plasmid pBAD30-*rsmS* was created as follows: oKA17 and oKA28 were used to amplify the *rsmS* ORF from Pba, this product was digested with *Eco*RI/*Hin*dIII and ligated with compatibly digested pBAD30 to create pBAD30-*rsmS*. The same procedure was used to create pBAD30-*priC* but with amplification of *priC* using oligonucleotides oKA89 and oKA91.

### Random transposon mutagenesis

Transposon mutagenesis was undertaken using the pDS1028 plasposon system^23^ or using the Tn*phoA*’2 transposon system^34,51^. Mutants bypassing *rsmB* repression of protease production were screened on caseinase agar (Nutrient broth, supplemented with 10 g l^−1^ Marvel skimmed milk powder and 1.5% agar). The *rsmB* mutant of Pcc39048 was identified by screening random transposon mutants for reduced protease production on caseinase agar as described previously^16^. Transposon insertions were transduced into different genetic backgrounds using the generalized transducing phage φM1 for Pba^52^ or φKP for Pcc39048^53^. Chromosomal transposon insertion sites were mapped by Random Primed PCR^54^ or by replicon cloning^23^.

### Phenotypic assays

Plate based assays assessing activities of PCWDEs were carried out by first normalizing the number of cells from an overnight culture for each strain to OD_600_ 1.0. Ten µl of normalised cells were spotted onto indicator plates and incubated at 25 °C for 48 hours. Cellulase and pectate lyase plates were prepared and developed as described previously^55^ and protease (gelatinase) plates were prepared and developed as described by Hankin and Anagnostaki^56^. Following development, haloes representing the respective enzyme activity were imaged. Swimming motility was assessed by spotting five µl of a normalized culture (OD_600_ = 0.1) onto a soft agar plate as described previously^9^. Carbapenem antibiotic production was monitored using an ESS biosensor seeded top lawn (0.7% agar). A normalized culture (OD_600_ = 1.0, five µl) of the strain tested was spotted onto the top lawn for live cell based assays, or a cut well assay was used^57^. For the cut well assay, culture supernatants were sterilized by passage through a 0.22 µm filter and were applied into standard sized wells of an ESS seeded top lawn. After incubation overnight at 30 °C, areas of haloes where ESS growth was inhibited surrounding each well were measured and normalized to OD_600_.

Liquid PCWDE assays were carried out on culture supernatants grown in PMB. At the indicated timepoint, a one ml sample of the culture was taken, separated by centrifugation at 16 000 *g*, the supernatant removed, snap frozen in liquid nitrogen and stored at −80 °C until assayed. Pectate lyase activity was determined by measuring the breakdown of polygalacturonic acid as described by Starr and colleagues^58^. Cellulase and protease activities of samples were determined using the methods described previously by Coulthurst *et al*.^59^ and Cubitt *et al*.^16^ respectively.

### Tuber virulence assays

All tuber virulence assays were performed using Maris Piper potatoes. Tubers were surface sterilized in 1% Virkon™ for five minutes and washed three times in sterile water. Each side of the sterilized tuber was then stabbed with a 200 µl sterile tip to create two inoculation sites. Overnight cultures of either the wild type or *rsmS* mutant were normalized so that 10^3^ cells were contained within 20 µl and this volume was inoculated into the potato into one of the sites. Then this process was repeated for the other strain at the second inoculation site. Each site of inoculation was sealed using sterile vacuum grease and the potato wrapped three times in damp paper towel. The potatoes were then incubated at 25 °C for four days. The weight of rotting tissue was then measured after removing the rotten area. Four biological replicates were performed and the average weight of rot compared for wild type Pba or an *rsmS* mutant.

### Acyl-homoserine lactone assays

Levels of OHHL were determined using an *E. coli* biosensor strain JM109 + pSB401 as described previously^25,59^. Culture supernatants were taken throughout growth and filtered by passing the sample through a 0.22 µm filter. One hundred µl of diluted (1:100) culture supernatants were mixed with 100 µl of JM109 + pSB401 cultures and incubated in black microtiter plates at 37 °C for three hours. Light emission (RLU) was measured using a Lucy Anthos luminometer (Pba) or a FLUOstar Omega plate reader (Pcc39048) and normalized to the original Pba culture OD_600_.

### Bioinformatics and Statistics

Alignments of nucleotide or amino acid sequences were performed using Clustal Omega^60^. Promoter regions were mapped using BPROM^61^. All amino acid searches were performed using PSI-BLAST^62^, conserved domains identified using Pfam^9^, and genomic locations were examined using existing tools on NCBI. The genome sequence of Pcc39048 used was Genbank accession number QHMC00000000^63^. Graphpad PRISM was used to perform statistical tests, as indicated in the text, and p-values were reported. Reported values represent the average of a minimum of three independent biological replicates, reported as n = 3.

## Supplementary information

Supplementary Information

## Data Availability

All data generated or analysed in this study are included in the manuscript.
