# Supplementary material for: The rsmS (ybaM) mutation causes bypass suppression of the RsmAB post-transcriptional virulence regulation system in enterobacterial phytopathogens
Source: Sci Rep. 2019 Mar 14;9:4525. doi: 10.1038/s41598-019-40970-3 (PMC6418279; doi:10.1038/s41598-019-40970-3)
Supplement: Supplementary file 1 — Supplementary Information [file 41598_2019_40970_MOESM1_ESM.pdf]

Supplemental Information

The *rsmS* (*ybaM*) mutation causes bypass suppression of the RsmAB post-transcriptional virulence regulation system in enterobacterial phytopathogens

Rita E Monson, Katinka Apagyi, Steve Bowden, Natalie Simpson, Neil Williamson, Marion F Cubitt, Steve Harris, Ian K Toth, George PC Salmond*

Supplemental Figure Legends

Supplemental Figure 1. **a**. Genomic context of the *priC* transposon insertion in strain MC3. An arrow with Tn above indicates the position of the transposon in this strain. **b**. Caseinase production on skimmed milk agar plates for wild type + pBAD30, *rsmB priC* + pBAD30, *rsmB priC* + pBAD30-*priC* and *rsmB priC* + pBAD30-*rsmS* after incubation at 25°C.

Supplemental Figure 2. Complementation of protease production by *rsmS* expressed from a plasmid. Normalized number of wild type, *rsmS*, *rsmB* or *rsmB, rsmS* strains carrying either pBAD30 or pBAD30-*rsmS* were spotted onto protease (gelatinase) indicator plates Amp (**a**) or Amp + 0.1% arabinose (**b**). Plates were developed after incubation at 25°C for 48 hours. Haloes surrounding colonies are indicative of enzyme production.

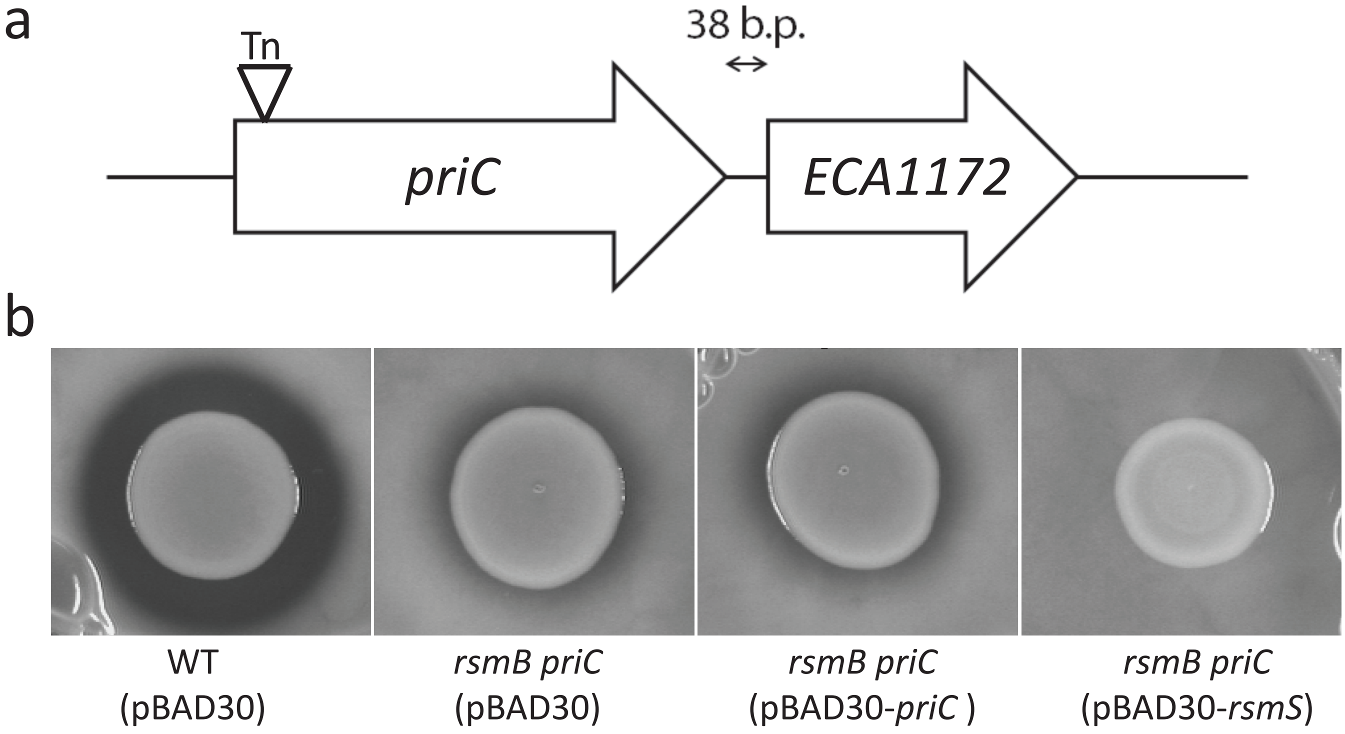

Supplemental Figure 1

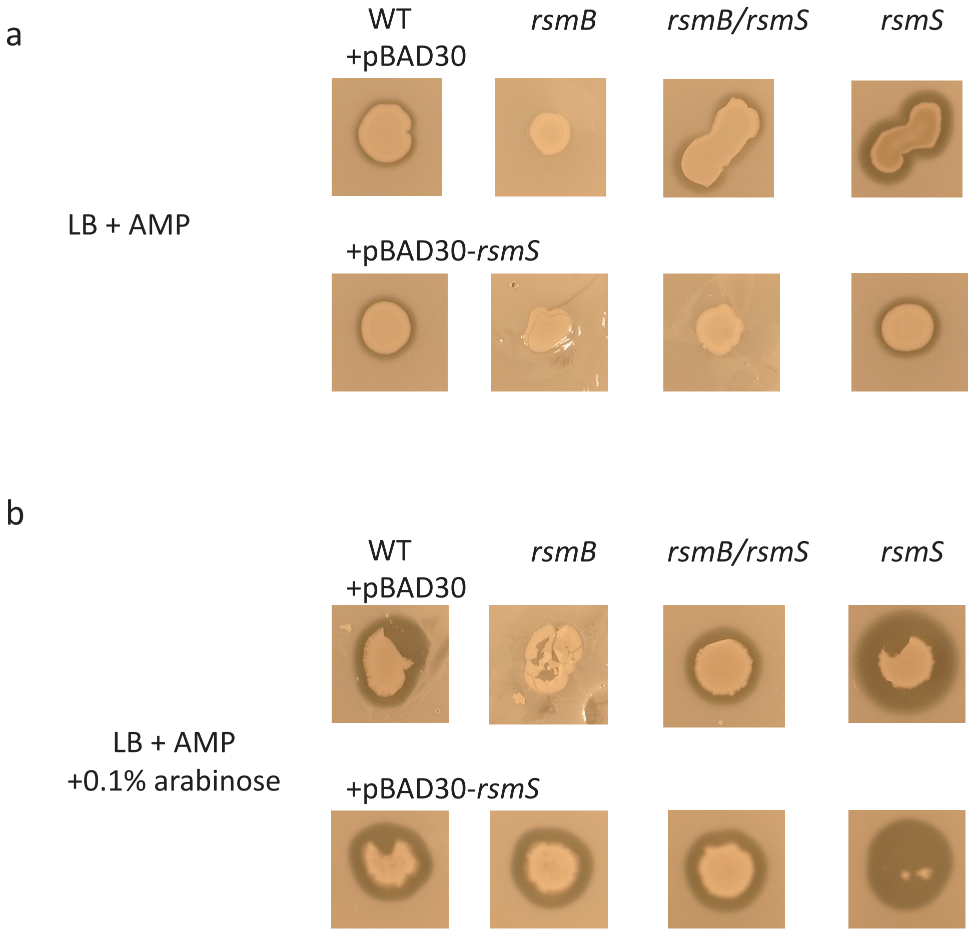

Supplemental Figure 2
